# Anxiety and Depression in the Portuguese Older Adults: Prevalence and Associated Factors

**DOI:** 10.3389/fmed.2017.00196

**Published:** 2017-11-20

**Authors:** Rute Dinis de Sousa, Ana Maria Rodrigues, Maria João Gregório, Jaime Da Cunha Branco, Maria João Gouveia, Helena Canhão, Sara Simões Dias

**Affiliations:** ^1^Chronic Diseases Research Center (CEDOC), EpiDoC Unit, NOVA Medical School, Universidade Nova de Lisboa (NMS/UNL), Lisbon, Portugal; ^2^EpiSaúde – Associação Científica, Évora, Portugal; ^3^Sociedade Portuguesa de Reumatologia, Lisbon, Portugal; ^4^Rheumatology Research Unit, Instituto de Medicina Molecular, Lisbon, Portugal; ^5^Faculdade de Ciências da Nutrição e Alimentação da Universidade do Porto, Porto, Portugal; ^6^Serviço de Reumatologia do Hospital Egas Moniz – Centro Hospitalar Lisboa Ocidental (CHLO-E.P.E.), Lisbon, Portugal; ^7^Promoting Human Potential Research Group, ISPA – Instituto Universitário, Lisbon, Portugal; ^8^Escola Nacional de Saúde Publica, Universidade Nova de Lisboa, Lisbon, Portugal; ^9^Escola Superior de Saúde do Instituto Politécnico de Leiria, Leiria, Portugal

**Keywords:** older, anxiety, depression, non-communicable chronic diseases, lifestyles, quality of life, function

## Abstract

Anxiety and depression in the elderly individuals have been studied around the world, and some authors consider them among the most serious problems faced by modern societies. With recent economic crisis—very important in Southern European countries—isolation, loneliness, and exclusion of the active society, mental problems are probably raising and associated with distinct factors. In this cross-sectional analysis, nested in a longitudinal population-based cohort study, we analyze anxiety and depression prevalence, and their related factors, in a representative cohort of Portuguese seniors. We used data retrieved from second wave of follow-up of EpiDoC Cohort—EpiDoC 2 study, which is composed by 10,661 adults, representative of adult Portuguese population. This study included all ≥65 years old EpiDoC 2 study participants, who responded to Hospital Anxiety and Depression Scale (HADS), *n* = 1,680. Sociodemographic, lifestyles, self-reported non-communicable diseases, health-related quality of life (EQ-5D-3D), physical function (HAQ), and health resources consumption data were collected. Anxiety and depression were assessed with HADS. Anxiety and depression prevalence were estimated. Multivariable logistic regression was used to assess anxiety and depression score determinants. The estimated prevalence of anxiety among Portuguese elderly is 9.6% and depression is 11.8%. Seniors with anxiety and seniors with depression have a higher probability to self-report higher levels of physical disability (OR = 3.10; 96% CI 2.12–4.52; OR = 3.08, 95% CI 2.29–4.14, respectively) and lower levels of quality of life (OR = 0.03, 95% CI 0.01–0.09; OR = 0.03, 95% CI 0.01–0.06, respectively). Female gender (OR = 2.77, 95% CI 1.53–5.00), low educational level (OR = 2.30, 95% CI 1.22–4.36), allergic (OR = 2.02, 95% CI 1.14–3.55), and rheumatic disease (OR = 2.92, 95% CI 1.74–4.90) were significantly and independently associated with the presence of anxiety symptoms. Physical inactivity (OR = 1.64, 95% CI 1.11–2.42) and low educational level (OR = 2.40, 95% CI 1.41–4.09) were significantly and independently associated with depression symptoms. Subjects that reported to drink alcohol daily or occasionally were negatively associated with depression symptoms. Anxiety and depression are frequent among Portuguese elderly. These prevalence rates suggest that preventing mental illness in senior population is a crucial need. A well-designed prevention strategy might have an effective action in raising the well-being of elderly.

## Introduction

Our world is now old and aging. Aging population is a long-term trend in Portugal, Europe, and around the World. According to United Nations data from 2015, the number of people over 65 has increased considerably in the largest regions of the world, and this aging is expected to accelerate in the coming decades (1). The same data point out that, in 2015, 1 in 8 people in the world were 60 years or older, totaling 901 million elderly people. A 176 million of these elderly people live in Europe.

In Portugal, the number of people over 65 years of age doubled in relation to the 1970s and, by 2015, was already over two million, with the population over 80 years old increasing fivefold. In concrete terms, there were 836,058 people aged 65 and over in Portugal in 1971. In 1977, they surpassed one million and, in 2012, two million. In 2015, they were 2,122,996 (2).

Although the epidemiological investigation has begun to converge regarding the estimation of the prevalence of anxiety and depression in elderly populations, there are still quite a few discrepancies (3). The sampling procedures diverge greatly: some studies use representative samples from the respective country, and others use samples of convenience. In addition, there is considerable instability in age cutoffs considered for the definition of the elderly or older adult. On the other hand, there is much variation in the operationalization of anxiety and depression, partly due to the use of different evaluation instruments. In fact, some authors consider that anxiety and depression are among the most serious problems faced by modern societies is depression among the elderly (4). Although depression is fairly common in the last years of life (5), there is great variation in its prevalence in studies worldwide (9–33%) (6).

Factors associated with aging, such as social isolation, reduced autonomy, financial insecurity, and poor health, cause an increase in the prevalence of these disorders (7).

Some studies point to psychosocial risk factors for anxiety disorders and late-onset depression: female gender, cognitive dysfunction, chronic illness, poor health perception, functional limitations, personality traits such as neuroticism and weak coping strategies (8, 9). Specifically, for anxiety disorder: not having children, low income, and experiencing traumatic events (10).

Given the social and economic context of our country, generating relevant evidence on anxiety and depressive symptoms in the Portuguese elderly population, and understanding them from a point of view that goes beyond the mental illness itself, by approaching the possible associated factors, is crucial. Data on elderly mental health and associated factors could be useful to address therapeutic programs and better planning of health care.

Therefore, to comprehend models of anxiety and depression independently in the Portuguese senior population, this study aims to: (1) determine the prevalence of anxiety and depression symptoms and (2) identify relevant associations between the symptoms of anxiety and depression and chronic self-reported non-communicable diseases, lifestyles, and sociodemographic characteristics.

## Materials and Methods

### Sample

To analyze the older adult Portuguese population in terms of symptoms of anxiety and depression, their association with non-communicable chronic diseases, function, lifestyles, and quality of life we used data from the second wave of follow-up for EpiDoC Cohort—EpiDoC 2 (CoReumaPt) study—done by computer-assisted telephone interviews (CATIs).

The EpiDoC 2 evaluation consisted on a structured questionnaire, applied through phone call interviews to 10,153 eligible participants of EpiDoC 1 (EpiReumaPt) (11–13), a large population-based sample, who consented to be contacted again for follow-up. The study population comprised adults (≥18 years old) living in the community, in Portugal Mainland and Islands (Azores and Madeira). Exclusion criteria were as follows: being resident in institutions and individuals unable to speak Portuguese or to complete the assessment protocol. Participants were selected through a process of multistage random sampling. The sample was stratified by region and population size.

**Figure 1:**
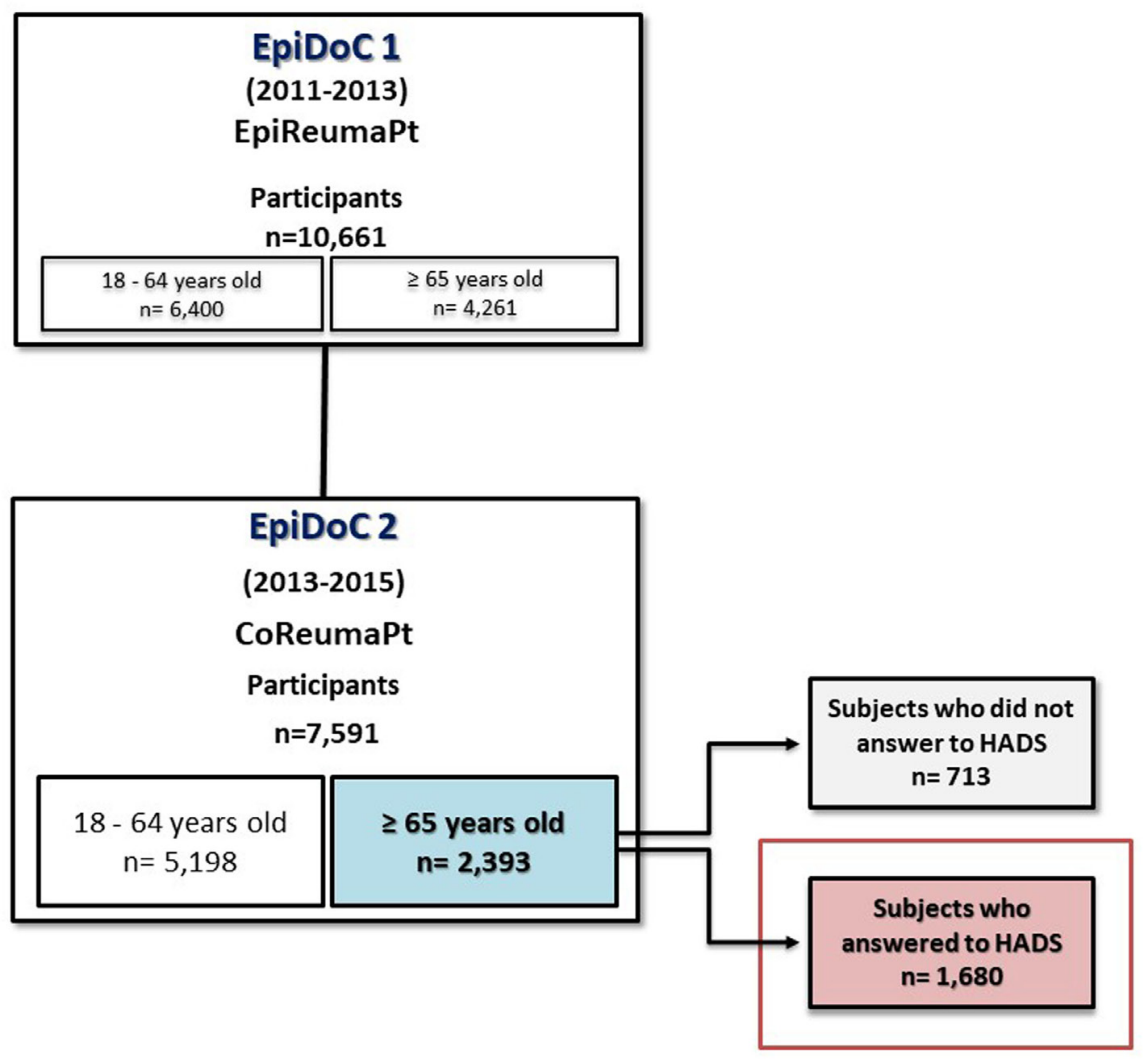
Flowchart describing population eligible for this study.

EpiDoC 2 (CoReumaPt) included 7,591 participants, representative of the adult Portuguese population. This study included 1,680 seniors (65 and more years old) of the EpiDoC 2 study, who responded to Hospital Anxiety and Depression Scale (HADS) (Figure 1).

### Data Collection

Data collection was performed from March 26th 2013 to July 27th 2015. A trained research assistants’ team was responsible for collecting the follow-up data from these subjects, by randomly call all the individuals. When a contact was not available, they would hold more attempts in different moments (morning, afternoon, evening, and weekends) to perform six attempts. The last contact had to have at least 1 month of interval from the previous one. Only then the contact would be abandoned. Rescheduling of the telephonic interviews to a more convenient moment was also an option. The interview was telephonically performed with the assistance of a CATI system.

Data were collected in a standardized form, and database access was protected by unique username and password, for each research team member, according to Portuguese Law of data collection (14).

### Measurements, Assessment, and Instruments

#### Case Definition

To evaluate symptoms of anxiety and depression in EpiDoC 2 study, the HADS Portuguese validated version was applied (15). The HADS was originally developed by Zigmond and Snaith (16) as a screening tool to apprehend clinically significant states of anxiety and depression in a non-psychiatric hospital setting. Individual anxiety and depression scores were calculated by summation of the appropriate seven items and thus can range from 0 to 21, with higher scores indicating higher levels of anxiety or depression, respectively. In both subscales, a score between 0 and 7 is “normal,” between 8 and 10 “mild,” between 11 and 14 “moderate,” and between 15 and 21 “severe” (idem). Presence of anxiety and depression symptoms was defined when HADS scale was ≥11, since Snaith suggested a score ≥11 was indicative of “caseness” to a mood disorder (17). We also used the HADS scale as a continuous outcome in the final analyses.

#### Sociodemographic and Socioeconomic Characteristics

Information on sociodemographic (sex, age, years of education, household composition, and NUT II), as well as socioeconomic variables (household income), was collected in the first wave—EpiDoC 1 study. Subjects were asked in the EpiDoC 2 study interview whether there have been changes.

#### Health Characteristics

In EpiDoC 1 study, individuals were asked if they had been previously diagnosed with some chronic disease (high cholesterol level, high blood pressure, rheumatic disease, allergy, gastrointestinal disease, cardiac disease, diabetes, thyroid and parathyroid disease, urolithiasis, pulmonary disease, hyperuricemia, cancer, neurologic disease, and hypogonadism), and this information was updated in the EpiDoC 2 study interview.

Self-reported height and weight were collected in EpiDoC 2. Based on these data, body mass index (BMI, weight/height^2^, in kg/m^2^) was calculated and categorized according to the World Health Organization classification in four categories: underweight (BMI < 18.5 kg/m^2^), normal (BMI between 18.5 and 24.9 kg/m^2^), overweight (BMI between 25 and 29.9 kg/m^2^), and obesity (BMI ≥ 30 kg/m^2^) (18).

Health-related quality of life was assessed using the Portuguese validated version of European Quality of Life questionnaire (EQ-5D-3L) (19, 20).

Function was evaluated based on the Portuguese version of Health Assessment Questionnaire (HAQ) (21, 22).

To assess health-care resources consumption, “number of medical appointments in the previous 12 months” and “hospitalization in the previous 12 months (yes/no)” was asked to participants.

#### Lifestyle Characteristics

In EpiDoC 2 study, questions concerning lifestyle habits included frequency of alcohol intake (daily, occasionally, and never), smoking habits (current smoker, past smoker, and never smoked), sleep habits (number of hours of sleep per day, categorized in <6 and ≥6 h/day). Physical activity level was classified based on the question related to the reported weekly frequency of physical activity. A frequency of once a week or more was considered “yes” to regular physical exercise.

### Statistical Analysis

#### Sample Weights

EpiDoC was designed to obtain a representative sample of the Portuguese population (13). To guarantee its representativeness, weighted proportions have, for this matter, been computed and are described elsewhere (13).

To maintain the representativeness of the sample in relation to the Portuguese population (Mainland and Islands) in the second wave, extrapolation weights were again computed and used in further statistical analysis. These were obtained by calibrating the extrapolation weights originally designed for the EpiDoC 1 study sample. We first compared the participants and non-participants of EpiDoC 2 study, concerning their sociodemographic, socioeconomic and rheumatic disease screening, and health status characteristics. Then, we decided to adjust weights based on the stratification by seven NUTII regions [Norte, Centro, Lisboa, Alentejo, Algarve, and islands (Azores and Madeira)], gender (male and female), age categories (18–35, 36–55, 56–65, ≥8–35, 36–55 years old for NUTII Norte, Centro, and Lisboa; 18–65, 66+ for NUTII Alentejo, Algarve, and islands), and rheumatic disease screening (positive/negative).

### Analysis and Statistical Software

Descriptive data for each categorical variable were presented as the absolute frequency and the correspondent proportion, weighted. The same adjustment has been done for the mean and SD for each continuous variable. Continuous variables were compared using Student’s *t*-test or ANOVA, and nominal variables were compared using chi-square test.

To assess the determinants of anxiety and depression symptoms, we first performed univariate analysis to approach relations between independent variables (non-communicable chronic diseases, lifestyles, and sociodemographic characteristics) and outcomes (presence of anxiety and depression symptoms). When univariate analyses resulted in *p*-value < 0.1, those variables were included into the multivariable logistic regression models. Multivariable models were constructed using a forward selection method. The following independent variables were tested: age, gender, NUTS II, education level (0–4 vs ≥5 years), BMI (underweight, normal, overweight, and obese), alcohol intake (never, daily, and occasionally), current smoking, physical activity (yes/no), and chronic non-communicable diseases—high blood pressure, diabetes, high cholesterol level, pulmonary disease, cardiac disease, neurologic disease, allergies, neoplasic disease, thyroid disease, hyperuricemia, and rheumatic disease. Age, gender, and NUTS II were forced to stay in the model. The goodness of fit of the final multivariable regression model for the outcome anxiety has *p* = 0.287, and the goodness of fit of the final multivariable regression model for the outcome depression has *p* = 0.4.

Significance level was set at 0.05. All analyses were weighted and executed using STATA IC version 12 (StataCorp. 2011. Stata Statistical Software: Release 12. College Station, TX, USA: StataCorp LP).

### Ethical Issues and Data Protection

EpiDoC 1 study was performed following the principles established by the Declaration of Helsinki (23) and revised in 2013 in Fortaleza. The study was reviewed and approved by the National Committee for Data Protection (Comissão Nacional de Proteção de Dados) and by the NOVA Medical School Ethics Committee. The participants provided informed consent to contribute in all phases of the study.

The EpiDoC 2 study was also approved by National Committee for Data Protection (in accordance with the Portuguese law number 67/98, October 26th, regarding protection of personal data) and was submitted to the same Ethics Committee. The study was conducted in accordance with the applicable laws and regulations including, but not limited to, the Guideline for Good Clinical Practice and the ethical principles stated in the Declaration of Helsinki (23).

Participants’ confidentiality is safeguarded by the nonexistence of identifiers on the database (only unique ID participants’ codes). Their names and contacts are stored separately from study data transmitted to the coordinating center. Thus, all data are kept anonymously and securely by the EpiDoC 2 study authorized staff. During EpiDoC 1 study, informed consent was also signed by those accepting to participate in the EpiDoC 2 study. There will be absolutely no disclosure of individual health information to the general public.

## Results

Our population of interest included 1,680 participants over 64 years old, 908 (54%) of those were females. Forty four percent were 75 years old or more. With respect to educational level, almost two-thirds of participants (*n* = 1,259; 76.03%) had less or equal to 4 years of education.

Most people with 65 years of age and more reported to live on a household income of less than 1,000€ per month; 35.8% reported the lowest income level of 500€/month. Moreover, elderlies tend to live in households are composed by only one (25.53%) or two persons (55.89%).

With respect to health-related characteristics, the observed mean number of non-communicable chronic diseases was 3.10 (±2.1). The most frequently reported chronic diseases were high blood pressure (57.3%), rheumatic diseases (50.53%), and high cholesterol level (49.7%).

People 65+ years old score EQ-5D-3L on average 0.61 (±0.77) for quality of life and 0.72 (±0.72) in HAQ for physical function.

The high consumption of health care resources by the older age group matches the health status. The average number of appointments was 7.74 (±8.40), and 24.16% was hospitalized in the last 12 months.

Regarding lifestyle habits, namely, in terms of alcohol intake and smoking habits, 36.43% of the individuals had reported a daily intake of alcohol beverages but only 5.39% were current smokers. Among the elderly, 43.65% of the subjects never consume alcohol and 66.35% of people aged 65 and above never smoked. Almost 60% of individuals above 65 years old do not engage in regular physical exercise, and around 70% of the Portuguese elderly sleep 6 or more hours per day.

### Prevalence of Anxiety and Depression Symptoms in the Elderly

This study found 176 elderly out of 1,680 (10.48%) presenting anxiety symptoms (HADS-A ≥11), corresponding to a prevalence of 9.59% when weighted to Portuguese elderly population (Table 1). The mean score for HADS-A was 5.04 (±3.74).

**Table 1:** Prevalence of anxiety and depression in the Portuguese senior population by age group.

	Elderly with anxiety symptoms (HADS-A ≥11)	Elderly with depression symptoms (HADS-D ≥11)

	*n* (%)	*n* (%)
Total	176 (9.59)	241 (11.77)
**Age group**		
65–69 y.o.	52 (12.38)	44 (7.29)
70–74 y.o.	45 (7.86)	65 (11.33)
75–79 y.o.	47 (8.99)	68 (15.16)
80–84 y.o.	20 (7.96)	42 (14.85)
≥85 y.o.	12 (9.56)	22 (1.68)

*%, weighted percentage; HADS-A, Hospital Anxiety and Depression Scale—Anxiety subscale; HADS-D, Hospital Anxiety and Depression Scale-Depression subscale; y.o., year old*.

In terms of depression symptoms, figures rise to 241 (14.34% in the present sample), equivalent to a prevalence of 11.77% of the Portuguese elderly population (Table 1). This population had a mean score for HADS-D of 5.27 (±4.07).

Sociodemographic, socioeconomic, lifestyle, and health-related characteristics of the Portuguese elderly population with and without anxiety symptoms are summarized in Table 2.

**Table 2:** Sociodemographic, socioeconomic, lifestyle, and health-related characteristics of the Portuguese elderly population with and without anxiety symptoms.

Sociodemographic and socioeconomic characteristics		Elderly without anxiety symptoms (HADS-A <11)	Elderly with anxiety symptoms (HADS-A ≥11)	*p*-Value	Adjusted *p*-value

		Mean (SD) or *n* (%)	Mean (SD) or *n* (%)		

		*n* = 1,504 (90.41%)	*n* = 176 (9.59%)		
Age* (years)		74.24 (6.60)	73.96 (6.91)	0.059	0.131
Sex*	Female	768 (83.85)	140 (15.15)	<0.001^†^	<0.001^†^
	Male	736 (95.44)	36 (4.56)		
NUTS II*	Norte	476 (89.88)	67 (10.12)	0.906	0.977
	Centro	378 (89.93)	49 (10.07)		
	Lisboa e Vale do Tejo	291 (90.56)	23 (9.44)		
	Alentejo	95 (92.11)	11 (7.89)		
	Algarve	57 (93.14)	3 (6.86)		
	Região Autónoma dos Açores	80 (93.04)	7 (6.96)		
	Região Autónoma da Madeira	127 (91.03)	16 (8.97)		
Education level*	≤4 years of education	1,103 (88.55)	156 (11.45)	0.001^†^	0.002^†^
	>4 years of education	377 (95.98)	20 (4.02)		
Household income* (€)	≤500	384 (89.15)	58 (10.85)	0.430	0.411
	501–750	301 (91.75)	31 (8.25)		
	751–1,000	152 (88.38)	16 (11.62)		
	>1,000	275 (94.52)	16 (5.48)		
Household composition	1	374 (88.89)	55 (11.11)	0.388	0.900
	≥2	1,130 (90.90)	121 (9.10)		
Anthropometric data*					
BMI	Underweight	9 (84.23)	2 (15.77)	0.806	0.723
	Normal	397 (91.5)	47 (8.5)		
	Overweight	647 (90.2)	59 (9.8)		
	Obese	297 (91.32)	40 (8.68)		
**Lifestyle characteristics**					
Alcohol intake*	Never	630 (86.3)	102 (13.7)	0.002^†^	0.054
	Daily intake	571 (94.7)	40 (5.5)		
	Occasionally	300 (90.6)	34 (9.4)		
Smoke*	Never	969 (89.5)	145 (10.5)	0.480	0.344
	Past	449 (91.2)	25 (8.8)		
	Present	85 (95.5)	6 (4.5)		
No regular physical exercise*	873 (91.0)	109 (9.0)	0.590	0.482
Sleep habits (h)	<6	250 (87.7)	38 (12.3)	<0.001^†^	0.003^†^
	≥6	586 (95.9)	31 (4.1)		
Quality of life and physical function**					
EQ5D (0–1)		0.66 ± 0.01	0.31 ± 0.02	<0.001^†^	<0.001^†^
HAQ score (0–3)		0.63 ± 0.03	1.4 ± 0.06	<0.001^†^	<0.001^†^
Number of non-communicable diseases*		2.92 ± 1.95	4.76 ± 2.33	<0.001^†^	<0.001^†^
High blood pressure (Y/N)		825 (87.8)	128 (12.2)	0.011^†^	0.021^†^
Diabetes (Y/N)		341 (90.8)	47 (9.2)	0.808	0.874
High cholesterol level (Y/N)		712 (89.3)	105 (10.8)	0.489	0.908
Pulmonar disease (Y/N)		154 (82.6)	35 (17.4)	0.080	0.028^†^
Cardiac disease (Y/N)		370 (87.6)	67 (12.4)	0.127	0.104
Gastrointestinal diseases (Y/N)		380 (86.4)	72 (13.6)	0.037^†^	0.121
Neurologic diseases (Y/N)		106 (84.1)	22 (15.9)	0.080	0.256
Allergies (Y/N)		313 (83.0)	57 (17.0)	0.011^†^	0.015^†^
Neoplasic disease (Y/N)		128 (91.2)	15 (8.8)	0.732	0.600
Thyroid disease (Y/N)		156 (80.9)	41 (19.1)	<0.001^†^	0.085
Hyperuricemia (Y/N)		193 (89.1)	27 (10.9)	0.655	0.194
Rheumatic disease (Y/N)		685 (84.0)	125 (16.0)	<0.001^†^	<0.001^†^
Health resources consumption**					
Was hospitalizes since last contact		343 (85.0)	60 (15.1)	0.016^†^	0.003^†^
Went to medical appointments in hospitals since last contact		7.79 ± 0.36	13.9 ± 4.4	0.150	0.043^†^

*%, weighted percentage; NUTS II, Nomenclatura das Unidades Territoriais, level II; BMI, body mass index (kg/m^2^); €, euro; HADS-A, Hospital Anxiety and Depression Scale—Anxiety subscale; HAQ, Health Assessment Questionnaire (0–3); EQ5D, European Quality of Life questionnaire five dimensions three levels (0–1)*.

*^†^p-Value ≤ 0.05*.

*p-Value was adjusted for age, sex, and region.

**p-Value was adjusted for age, sex, region, and number of non-communicable diseases.

Table 3 summarizes sociodemographic, socioeconomic, lifestyle, and health-related seven characteristics of the Portuguese elderly population with and without depression symptoms.

**Table 3:** Sociodemographic, socioeconomic, lifestyle, and health-related characteristics of the Portuguese elderly population with and without depression symptoms.

Sociodemographic and socioeconomic characteristics		Elderly without depression symptoms (HADS-D <11)	Elderly with depression symptoms (HADS-D ≥11)	*p*-Value	Adjusted *p*-value
				
		Mean (SD) or *n* (%)	Mean (SD) or *n* (%)		
				
		*n* = 1,439 (88.23%)	*n* = 241 (11.77%)		
Age* (years)		73.57 (6.51)	75.74 (7.23)	<0.001^†^	0.001^†^
Sex*	Female	747 (84.47)	161 (15.53)	<0.001^†^	<0.001^†^
	Male	692 (91.64)	80 (8.36)		
NUTS II*	Norte	463 (87.84)	80 (12.16)	0.020^†^	0.084
	Centro	360 (85.41)	67 (14.59)		
	Lisboa e Vale do Tejo	281 (93.63)	33 (6.37)		
	Alentejo	89 (87.2)	17 (12.8)		
	Algarve	50 (86.89)	10 (13.11)		
	Região Autónoma dos Açores	76 (87.77)	11 (12.23)		
	Região Autónoma da Madeira	120 (85.62)	23 (14.38)		
Education level*	≤4 years of education	1,046 (65.37)	213 (34.63)	<0.001^†^	<0.001^†^
	>4 years of education	373 (76.84)	24 (23.16)		
Household income* (€)	≤500	342 (80.53)	100 (19.47)	<0.001^†^	0.007^†^
	501–750	291 (88.06)	41 (11.94)		
	751–1,000	154 (96.26)	14 (3.74)		
	>1,000	271 (93.22)	20 (6.78)		
Household composition	1	347 (84.05)	82 (15.95)	0.009^†^	0.161
	≥2	1,092 (89.58)	159 (10.42)		
Anthropometric data*					
BMI	Underweight	9 (74.53)	2 (24.47)	0.293	0.245
	Normal	388 (89.99)	56 (10.01)		
	Overweight	620 (89.98)	86 (10.02)		
	Obese	284 (86.98)	53 (13.02)		
**Lifestyle characteristics**					
Alcohol intake*	Never	579 (83.2)	153 (16.8)	<0.001^†^	0.001^†^
	Daily intake	553 (91.7)	58 (8.3)		
	Occasionally	304 (92.1)	30 (7.9)		
Smoke*	Never	926 (85.8)	188 (14.2)	0.001^†^	0.154
	Past	434 (93.0)	40 (7.0)		
	Present	78 (90.2)	13 (9.8)		
No regular physical exercise*		807 (85.0)	175 (15.0)	<0.001^†^	0.001
Sleep habits (h)	<6	241 (86.1)	47 (13.9)	0.008^†^	0.048^†^
	≥6	528 (92.1)	59 (7.9)		
Quality of life and physical function**					
EQ5D (0–1)		0.67 ± 0.02	0.32 ± 0.02	<0.001^†^	<0.001^†^
HAQ score (0–3)		0.61 ± 0.03	1.38 ± 0.59	<0.001^†^	<0.001^†^
Number of non-communicable diseases		2.95 ± 1.93	4.36 ± 2.70	<0.001^†^	<0.001^†^
High blood pressure (Y/N)*		804 (87.2)	149 (12.76)	0.093	0.121
Diabetes (Y/N)*		315 (84.8)	73 (15.2)	0.039^†^	0.022^†^
High cholesterol level (Y/N)*		688 (86.0)	129 (14.0)	0.013^†^	0.056
Pulmonar disease (Y/N)*		150 (82.9)	39 (17.1)	0.023^†^	0.038^†^
Cardiac disease (Y/N)*		347 (83.6)	90 (16.5)	0.001^†^	0.015^†^
Gastrointestinal diseases (Y/N)*		367 (63.6)	85 (36.4)	0.019^†^	0.019^†^
Neurologic diseases (Y/N)*		102 (80.5)	26 (19.5)	0.028^†^	0.009^†^
Allergies (Y/N)		312 (87.9)	58 (12.1)	0.074	0.970
Neoplasic disease (Y/N)*		122 (87.3)	21 (12.7)	0.686	0.676
Thyroid disease (Y/N)*		157 (80.6)	40 (19.4)	0.001^†^	0.061
Hyperuricemia (Y/N)*		181 (83.2)	39 (16.8)	0.010^†^	0.009^†^
Rheumatic disease (Y/N)*		649 (82.9)	161 (17.1)	<0.001^†^	<0.001^†^
Health resources consumption**					
Was hospitalizes since last contact		334 (86.6)	69 (13.4)	0.226	0.043^†^
Went to medical appointments in hospitals since last contact		8.170 ± 0.634	8.961 ± 1.092	0.455	0.376

*%, weighted percentage; NUTS II, Nomenclatura das Unidades Territoriais, level II; BMI, body mass index; €, euro; HADS-D, Hospital Anxiety and Depression Scale—Depression subscale; HAQ, Health Assessment Questionnaire (0–3); EQ5D, European Quality of Life questionnaire five dimensions three levels (0–1)*.

*^†^p-Value ≤ 0.05*.

*p-Value was adjusted for age, sex, and region.

**p-Value was adjusted for age, sex, region, and number of non-communicable diseases.

### Anxiety and Depression Symptoms and Physical Function in Seniors

Seniors with anxiety have a higher probability to have reported, even after the adjustment for sex, age, region, and number of non-communicable diseases, higher levels of physical disability (OR = 3.10; 96% CI 2.12–4.52).

Similar results were found for depression symptoms and seniors with depression have reported higher levels of physical disability (OR = 3.08, 95% CI 2.29–4.14).

### Anxiety and Depression Symptoms and Quality of Life in the Elderly

In terms of quality of life (EQ-5D-3L score), seniors with anxiety have a higher probability to report lower levels of quality of life (OR = 0.03, 95% CI 0.01–0.09). These results were found after the adjustment for sex, age, region, and number of non-communicable diseases.

Multivariable analyses adjusting to sex, age, region, and number of non-communicable diseases also showed that seniors with depression symptoms have a higher probability to have reported lower levels of quality of life (OR = 0.03; 95% CI 0.01–0.06).

### Anxiety and Depression Symptoms and Health-care Resources Consumption in the Elderly

Logistic regression adjusted to sex, age, region, and number of non-communicable diseases stressed that seniors with anxiety symptoms have a higher probability to have reported higher number of hospitalizations in the previous 12 months (OR = 2.53, 95% CI 1.37–4.67) and have reported a higher number of medical appointments in the previous 12 months.

Seniors with depression symptoms have also a higher probability to have reported higher number of hospitalizations in the previous 12 months (OR = 1.55, 95% CI 1.01–2.35). In terms of number of medical appointments in the previous 12 months, there is no significant difference between elderly with and without depressive symptoms.

### Sociodemographic, Lifestyles, and Health Factors Independently Associated with Anxiety in Seniors

Multivariable logistic regression, including all previous significant independent variables, showed that being a woman (OR = 2.77, 95% CI 1.53–5.00) and have a low educational level (OR = 2.30, 95% CI 1.22–4.36) were significantly associated with anxiety symptoms. Regarding self-reported non-communicable chronic diseases, our results showed that only allergies (OR = 2.02, 95% CI 1.14–3.55) and rheumatic disease (OR = 2.92, 95% CI 1.74–4.90) were statistically significant associated with anxiety symptoms (Table 4).

**Table 4:** Factors associated with anxiety symptoms in seniors.

	Anxiety symptoms (yes/no)						
	Univariable logistic regression			Multivariable logistic regression		
	OR	95% CI	*p*-Value	OR	95% CI	*p*-Value
**Gender**						
Male	Ref.					
Female	3.74	2.12–6.62	0.000	2.77	1.53–5.00	0.001
Age (years)	0.98	0.94–1.02	0.371	0.96	0.92–1.00	0.078
**NUTS II**						
Norte	Ref.			Ref.		
Centro	0.99	0.63–1.57	0.982	0.98	0.60–1.60	0.939
Lisboa	0.92	0.29–3.00	0.897	0.97	0.37–2.54	0.949
Alentejo	0.76	0.37–1.57	0.462	0.79	0.36–1.73	0.553
Algarve	0.65	0.18–2.42	0.525	0.93	0.23–3.78	0.918
Azores	0.66	0.28–1.58	0.356	1.14	0.41–3.18	0.796
Madeira	0.87	0.46–1.66	0.683	0.91	0.48–1.75	0.785
Live alone (Y/N)	1.25	0.75–2.07	0.389			
**Level of education**						
≤4 years	3.09	1.72–5.54	0.000	2.30	1.22–4.36	0.010
>4 years	Ref.					
**BMI**						
Underweight	Ref.					
Normal	0.50	0.10–2.45	0.390			
Overweight	0.58	0.11–3.05	0.521			
Obese	0.51	0.10–2.52	0.407			
**Alcohol intake**						
Never	Ref.					
Daily intake	0.35	0.20–0.62	<0.001			
Occasionally	0.65	0.33–1.30	0.224			
**Smoke**						
Never	Ref.					
Past	0.82	0.33–2.06	0.680			
Present	0.40	0.15–1.06	0.066			
No regular physical exercise	0.85	0.48–1.53	0.590			
High blood pressure (Y/N)	2.03	1.18–3.50	0.011			
Diabetes (Y/N)	0.94	0.56–1.58	0.808			
High cholesterol level (Y/N)	1.23	0.69–2.20	0.489			
Pulmonar disease (Y/N)	1.64	0.94–2.85	0.080			
Cardiac disease (Y/N)	1.50	0.89–2.52	0.127			
Gastrointestinal diseases (Y/N)	1.80	1.03–3.13	0.039			
Neurologic diseases (Y/N)	1.85	0.93–3.67	0.080			
Allergies (Y/N)	2.43	1.23–4.79	0.011	2.02	1.14–3.55	0.015
Neoplasic disease (Y/N)	0.89	0.46–1.72	0.732			
Thyroid disease (Y/N)	2.51	1.50–4.20	<0.001			
Hyperuricemia (Y/N)	1.13	0.65–1.98	0.655			
Rheumatic disease (Y/N)	4.36	2.65–7.16	<0.001	2.92	1.74–4.90	<0.001

*HADS-A, Hospital Anxiety and Depression Scale—Anxiety subscale; OR, odds ratio; CI, confidence interval; HAQ, Health Assessment Questionnaire (0–3); EQ5D, European Quality of Life questionnaire five dimensions three levels (0–1); BMI, body mass index*.

*^†^*p*-Value ≤ 0.05; GoF (*p* = 0.287)*.

### Sociodemographic, Lifestyles, and Health Factors Independently Associated with Depression in Seniors

All previous significant independent variables were included in a multivariable logistic regression to depression symptoms. In this analysis, low educational level (OR = 2.40, 95% CI 1.41–4.09), physical inactivity (OR = 1.64, 95% CI 1.11–2.42), and alcohol intake on a daily (OR = 0.54, 95% CI 0.35–0.83) and occasionally basis (OR = 0.49, 95% CI 0.29–0.84) were protectors of depression (were associated with low risk of depression symptoms) (Table 5).

**Table 5:** Factors associated with depression symptoms in seniors.

	Depression symptoms (yes/no)						
	Univariable logistic regression			Multivariable logistic regression		
	OR	95% CI	*p*-Value	OR	95% CI	*p*-Value
**Gender**						
Male	Ref.					
Female	2.02	1.42–2.85	<0.001	1.30	0.87–1.94	0.207
Age	1.05	1.02–1.07	<0.001	1.02	1.00–1.05	0.064
**NUTS II**						
Norte	Ref.					
Centro	1.23	0.84–1.82	0.289	1.11	0.73–1.70	0.607
Lisboa	0.49	0.29–0.84	0.009	0.51	0.28–0.96	0.037
Alentejo	1.06	0.58–1.94	0.849	1.18	0.64–2.18	0.603
Algarve	1.09	0.49–2.44	0.834	0.25	0.55–2.83	0.590
Azores	1.01	0.49–2.04	0.985	0.56	0.75–3.26	0.237
Madeira	1.21	0.70–2.11	0.492	1.20	0.66–2.18	0.547
Live alone (Y/N)	1.63	1.13–2.35	0.009			
**Level of education (years)**						
≤4	3.57	2.16–5.92	<0.001	2.40	1.41–4.09	0.001
>4	Ref.					
**BMI**						
Underweight	Ref.					
Normal	0.32	0.06–1.74	0.189			
Overweight	0.33	0.06–1.72	0.186			
Obese	0.44	0.08–2.30	0.334			
**Alcohol intake**						
Never	Ref.					
Daily intake	0.45	0.30–0.66	<0.001	0.54	0.35–0.83	0.005
Occasionally	0.42	0.26–0.70	0.001	0.49	0.29–0.84	0.009
**Smoke**						
Never	Ref.					
Past	0.46	0.30–0.70	<0.001			
Present	0.66	0.30–1.44	0.300			
No regular physical exercise	2.07	1.44–2.98	<0.001	1.64	1.11–2.42	0.013
High blood pressure (Y/N)	1.35	0.95–1.92	0.094			
Diabetes (Y/N)	1.50	1.02–2.21	0.040			
High cholesterol level (Y/N)	1.53	1.09–2.14	0.014			
Pulmonar disease (Y/N)	1.71	1.07–2.73	0.024			
Cardiac disease (Y/N)	1.83	1.27–2.64	0.001			
Gastrointestinal diseases (Y/N)	1.56	1.07–2.27	0.020			
Neurologic diseases (Y/N)	1.97	1.06–3.63	0.031			
Allergies (Y/N)	1.07	0.72–1.57	0.741			
Neoplasic disease (Y/N)	1.12	0.65–1.94	0.686			
Thyroid disease (Y/N)	2.01	1.31–3.08	0.001			
Hyperuricemia (Y/N)	1.74	1.14–2.65	0.011			
Rheumatic disease (Y/N)	2.94	2.04–4.23	<0.001	2.12	1.45–3.09	<0.001

*HADS-D, Hospital Anxiety and Depression Scale-Depression subscale; OR, odds ratio; CI, confidence interval; HAQ, Health Assessment Questionnaire (0–3); EQ5D, European Quality of Life questionnaire five dimensions, three levels (0–1); BMI, body mass index*.

*Household composition: number of people that live in the house of the participant, including himself/herself*.

*^†^*p*-Value ≤ 0.05; GoF (*p* = 0.04056)*.

## Discussion

This study showed a prevalence of 9.6% of anxiety symptoms among the Portuguese with 65 or more years old. This number is lower than the ones found in international studies over Australia, European, and American countries (24). These authors referred prevalence of significant anxiety symptoms between 15 and 52% in the elderly living in the community. Almost 10 years ago, an epidemiological study in Portugal, whose data were collected between 2008 and 2009, found the prevalence of generalized anxiety disorder in people over 65 years of age at 2.6% and the prevalence of any anxiety disorders 20% for the same age group (25).

Regarding depression, the estimated prevalence is of 11.8% for the Portuguese elderly living in the community, in line with the result of 12% for Major Depressive Disorder of the National Epidemiologic Study of Mental Health (25). In the Netherlands, however, depressive symptoms had a prevalence of 24% for older adults—55 years old and older (26), and Castro-Costa et al. exposed a prevalence of depressive symptoms ranging from 33% in France, Italy, and Spain, to 18–19%, in Sweden, Denmark, Netherlands, Germany, Austria, and Switzerland in adults over 50 years old (6).

Although the presented figures may suggest that Portuguese elderly are less anxious and depressed than other peoples in Europe and around the globe, one should be very careful when comparing these numbers. There are core discrepancies regarding methods of data collection, different instruments of assessing depression and anxiety, different nosological entities considered (major depression disorder, minor depression disorder, generalized anxiety disorder vs symptoms of depression and anxiety), and finally different age groups cutoffs (≥50 and ≥55 vs ≥65).

As Byrne stressed differences in anxiety prevalence rates may be due to differences among populations, but also to differences in assessment tools and algorithms used for diagnosis such as State Trait Anxiety Inventory and Anxiety Disorder Scale (27). Some studies use the hierarchical approach to diagnosis, which may lead to lower prevalence by excluding individuals with diagnostic criteria that also have criteria for diagnosing other higher disorders in the hierarchy (3).

Our study demonstrates a close relationship between physical function and anxiety and depression showing that seniors with lower physical function are more likely to report anxiety and/or depression symptoms. Lenze and collaborators performed a literature review on the association of late life depression and anxiety with physical disability. Their findings indicated that depression was a risk factor for disability, but also in the other way, being disability a risk factor for depression (28). Brenes and colleagues studied the influence of anxiety on the progression of physical disability in a community-based observational work, concluding that, after adjusting for confounders, anxiety continued to predict the development of disability in activities of daily living (29).

In terms of quality of life, our results suggest that seniors with anxiety and/or depression are more likely to have reported lower levels of quality of life. Sivertsen and colleagues, in their review article of 74 studies, concluded the same, demonstrating that depressed older people had poor global quality of life than non-depressed individuals. They added that this association was stable over time and independently of how quality of life was measured (30). Brenes also considered anxiety in relation to quality of life, and her conclusions were similar: anxiety was significantly associated with all domains of quality of life, and as severity of anxiety symptoms increased, quality of life decreased (31). Having very low income was also related to higher depression. This is in line with what Valvanne and collaborators defended in their elderly population-based study in Finland (32). McCall and colleagues findings in USA also corroborated the conclusions that related low income to higher scores of depression (33).

To assess determinants for anxiety and depression, a multivariable logistic regression, using socioeconomic factors and chronic diseases was developed.

The results considering the outcome as a binary variable “Anxiety disorders (Yes/No)” showed that being a woman, having less than 5 years of education, having a rheumatic disease and allergies were factors highly associated to higher scores of the anxiety diagnosis in Portuguese elders. Beekman and colleagues had already pointed sex and education level out as relevant to understand anxiety in their study with LASA cohort data (34).

Although other studies found a significant higher rate of anxiety in women with 55 or more years old (35), this was not found in our population.

A global model using multivariable logistic regression was also used to capture the framework of factors related to depression in Portuguese elderly, level of education, physical inactivity, alcohol intake on a daily and occasionally basis, and rheumatic disease should be taken into account when considering depression in this population.

This is in line with the literature supporting that physical activity is associated with lower levels of depression (36) Also, several studies suggest that lower education is associated with depression (37–39).

Regarding alcohol intake, the results found denote that the daily or occasional consumption of alcoholic beverages decreases the probability of reporting depression among seniors. Although this result can be unsettling at first glance, it is important to retain that this study assessed consumption frequency, rather than type or quantity. In the literature, several studies associate alcohol misuse or alcoholism with higher levels of depression (40, 41). That is, however, a different matter.

Although literature often expresses a relationship between anxiety and/or depression and diabetes (42), pulmonary (43), and neoplasic diseases (44), these associations were not confirmed in Portuguese senior population. Our study confirms, however, conclusions of previous studies on rheumatic diseases (11, 45, 46) stressing their important association with both anxiety and depression, as well as the relationship between allergies and anxiety, patent in the literature (47).

Interestingly, the female sex is also described in literature as an important determinant of depression (46, 48). In our study, it was strongly significant even with multivariable analyses with single factors separately, such as non-communicable chronic diseases, quality of life, or function. But within a global model including all significant variables for depression, it loses its previous relevance.

Regarding age, Alameda County Study demonstrated a raise of depression prevalence with age (49). But multivariable analyses showed that the effect of age was related to physical health problems and disability associated with aging. Several studies concluded the same way, indicating that although depression prevalence increases with age, it should be due to age-related factors than age itself (50, 51).

Study limitations should be considered when interpreting results. Being cross-sectional, this analysis does not allow to draw conclusions about the effects of causality between the variables and the chronological order of the events.

Although Breeman defended HADS being used for clinical and non-clinical population (52), the use of a specific instrument to assess anxiety and depression in this particular age group is arguable.

Despite of what has been referred, several strengths related to both internal and external validity should be pointed out. Data come from large, nationally representative sample of the Portuguese elder population followed since 2011, allowing its generalization for the age group in our country. It should also be noted that data were collected by a team of 10 interviewers who were extensively and properly trained, whose proceedings were standardized, and regular and rigorously monitored to assess quality and reduce bias.

In conclusion, key findings of this study are as follows: (1) Portuguese elderly population have prevalence of anxiety and depression around 10 and 12%, respectively; (2) anxiety and depression are associated to different factors; (3) health-related quality of life and physical function play an important role in depression and anxiety; and (4) level of education distinguishes senior in terms of anxiety and depression.

## Ethics Statement

This study was carried out in accordance with the recommendations of Guidelines for Good Clinical Practice and the ethical principles stated in the Declaration of Helsinki, National Committee for Data Protection (Comissão Nacional de Proteção de Dados) and by the NOVA Medical School Ethics Committee, with written informed consent from all subjects. All subjects gave written informed consent in accordance with the Declaration of Helsinki. The protocol was approved by the National Committee for Data Protection (Comissão Nacional de Proteção de Dados) and by the NOVA Medical School Ethics Committee.

## Author Contributions

RS conducted the study and wrote the paper. AR and MGr supported the development of the study design and methodology and reviewed the paper. JB is co-responsible for the EpiDoC Cohort and reviewed the paper. MGo and HC supported the whole development of the study and writing and reviewed the paper. SD supported the data analysis process.

## Conflict of Interest Statement

The authors declare that the research was conducted in the absence of any commercial or financial relationships that could be construed as a potential conflict of interest.
